# Supplementary material for: A lysine ring in HIV capsid pores coordinates IP6 to drive mature capsid assembly
Source: PLoS Pathog. 2021 Feb 1;17(2):e1009164. doi: 10.1371/journal.ppat.1009164 (PMC7850482; doi:10.1371/journal.ppat.1009164)
Supplement: Attachment — Submitted filename: K25 Response to Reviewers_Final.docx [file ppat.1009164.s007.docx]

**Part II – Major Issues: Key Experiments Required for Acceptance**

Reviewer #1

Q1. Figure 1: While IP5 is more practical to be able to obtain a crystal structure, it is unclear whether it really can substitute for IP6. Can the authors minimally show the model of IP6 in this pore and demonstrate that there is no steric hindrance to accommodating an IP6 molecule in that pocket given the axial vs. equatorial orientation of the phosphates? This point should also be clearly discussed in the text.

A1. We have modelled IP6 into our IP5 structure as suggested (Fig S1A). We have inserted the following into the text:

Lines 136-140: “Based on this structure and a previous structure of hexamer with one bound IP6 (6ES8), we modelled a second IP6 into the pore (Fig S1A). The axial phosphates of both molecules are readily accommodated without steric hindrance. While our model depicts a planar conformation, molecular dynamics simulations suggest that two IP6 molecules can also be accommodated when they adopt non-planar orientations.”

Q2. I think the authors should at least aim to separate whether the K25A mutation destabilizes the CA lattice because of the inability to bind IP6 vs. an intrinsic defect in assembly/stability. While I see that this is a difficult chicken-egg type of problem, inclusion of other mutants/controls will strengthen the manuscript and help address this problem. Otherwise, K25 is just another amino acid (in addition many that have already been described in the field) that when mutated destabilizes the core. Thus, I propose:

a. Figure 3a should be expanded to include R18G mutation as a positive control as well as R18G/K25A double mutant (as perhaps the effect will be more apparent once the R18 is mutated?). Other CA destabilizing mutants (i.e. P38A and K203A) should be included as negative controls. In addition, K25A is a pretty drastic mutation and other substitutions such as K25N and K25Q may help dissect the effects on IP6 coordination from the effects on core destabilization.

A2a. We thank the reviewer for this suggestion and have included new data on R18G , R18G/K25A and K25N in Figure 3a. Mutants that stabilize or destabilize the hexamer may alter its affinity for IP6 indirectly (because the hexamer is required for IP6 binding) and so we have omitted these in the present work, which is focused on K25 as a direct IP6 binding residue.

Q2b. Figure 4 should be expanded to include less drastic K25 substitutions.

A2b. We chose two mutants for this experiment. K25R is the least drastic substitution, as it maintains the positive charge. Other substitutions would change the physicochemical properties at this position. The other mutant we chose K25A, employs a classic alanine substitution that is widely-used in the protein-protein interaction field because it removes native interactions at a position without introducing new positive or negative interactions that don’t exist in the wild-type protein.

Q2c. Figure 6 should be expanded to include less drastic K25 substitutions as well as K203A or P38A substitutions.

A2c. We thank the reviewer for this suggestion and have included K25N and P38A in Figure 6. P38A behaves similarly to wild-type in that it readily assembles with either NaCl and IP6. K25N behaves like K25A and shows impaired assembly with IP6.

Q3 How do the authors reconcile the seemingly opposite conclusions from Figure 3 and Figure 6? What is the relevance of conditions tested in Figure 6 for HIV-1 biology? How would the result look if a different IP6 concentration was chosen?

A3. The Cryo-ET data in Figure 3C shows that K25A has a mature assembly defect. The in vitro assembly data in Figure 6 supports this conclusion by showing that K25A is unable to assemble efficiently. The nanoDSF data in Figure 3A shows that binding of IP6 still occurs to K25A, however the hexamers in these experiments are stabilized with disulphide bonds. This likely explains why mutating K25 did not have an effect whereas it does in our in vitro and in virion capsid assembly experiments. This was unclear in the previous manuscript and we have taken the opportunity to introduce this as a possibility earlier in the manuscript when presenting the nanoDSF data (Lines 194-196, 201-202). We have also directly compared these different datasets in the discussion (e.g. Lines 364-366). Figure 6 uses IP6 concentrations that are higher than those found in the cell, however IP6 is significantly enriched by HIV during budding (~ 1 mM). We agree it is important to show that assembly is possible even at lower IP6 concentrations and so we have included EM data showing cones can form at 10 µM IP6 (Fig S6 B).

Q4. Results from Figure 3e have inconsistencies. How come R18G can increase the GFP signal at similar levels as K25R?

A4. The abrogation assay in Figure 3e is a confusing experiment and we apologize that it was not explained clearly in the text. We have amended this to introduce clarity. Importantly, in each experiment a WT GFP virus is mixed with one of a number of variant RFP viruses. The dose of WT GFP virus is kept the same while the RFP virus dose is increased. So, in the case of the WT-GFP + R18G-RFP experiment referred to, adding an increasing dose of the R18G-RFP virus increasingly titrates out TRIM5, counteracting its restriction. This leads to an increase in WT-GFP infection because fewer virions are being restricted by TRIM5. One might expect more R18G-RFP infection as well, because more virus is being added, but although the R18G-RFP virus is competent to bind and saturate TRIM5 it is not capable of undergoing reverse transcription and so no increase in RFP signal is measured. In contrast, K25R is both infectious (albeit less than WT) and capable of saturating TRIM5 resulting in an increase of both GFP and RFP signals. We have added the following lines to clarify these points:

Lines 218-221: “We co-infected cells with a consistent dose of WT virus that encodes a GFP reporter gene and a variety of mutant viruses that encode an RFP reporter gene (for instance, a single infection includes both WT-GFP and K25R-RFP).”

Lines 233-236: “R18G-RFP virus had a similar ability to saturate TRIM5 as K25R, but R18G-RFP infectivity was drastically lower compared to K25R (Fig 3D, RFP virus). This suggests R18G viruses form higher order capsid assemblies that are largely non-infectious. This is consistent with previous data showing that R18G can form cores but is unable to reverse transcribe in cells [2, 10, 19].“

Reviewer #2:

Q1. My major concern is about unifying the initial and final experiments regarding the role of IP6 in viral particle formation. If the authors hypothesis is correct and IP5-6 should be bound to K25 to enable maturation, the experiments with purified capsid cores would never work as IP5-6 is added a posteriori to a sample that lacks capsid assemblies (as authors nicely showed). Thus, IP5-6 might be present in the viral particle while this is being formed at the membrane of the cell to enable posterior capsid core assembly during maturation. I wonder if there is any way to test this hypothesis by temporary increasing the levels of IP6/5 in cells (e.g. by overexpression of the IP kinases or by transfection/electroporation of IP6/5?). In this scenario, maybe the proportion of defective particles found in both WT, K25R and/or K25A mutant would be altered (Fig. 3c). Similarly, depletion of IP6/5 in cells should cause an increase of defective particles with Wt virus. Either way, I think these results should be better explained in the text, so the potential interpretation(S) and derived working models and hypotheses are clearer for a non-expert readers.

A1. It is true that IP6 is present in the viral particle, where it is recruited and enriched by K158 & K227 in immature hexamers (Dick, 2018; Mallery 2018). This leads to a high local IP6 concentration within the virion (~ 1 mM) and, crucially, means it is available to stabilize mature hexamers during maturation. However, the data in the present manuscript suggest that mutants of K25 are unable to coordinate IP6 effectively and this leads to a defect in maturation and a substantial reduction in infectivity. Importantly, while maturation efficiency is greatly reduced, a low fraction of K25 mutant virions within the population still form correctly assembled capsids as evidenced by the fact that K25A and K25R still display some infectivity (albeit much lower than WT). This explains why we are able to recover some purified K25 mutant cores from virions for encapsidated reverse transcription assays. The proposed experiments modulating cellular IP5/6 levels are a great suggestion but unfortunately this does not change IP5/6 levels packaged in the virion. Previously, we have shown that modulating cellular IP5/6 levels by changing kinase expression alters the number of viruses produced but not the number of IP5/6 molecules per virion (Mallery, 2019). We also fully take the point that clarification was needed to explain the significance of our different datasets to the two models proposed for K25 – capsid assembly and nucleotide import. We have therefore added the following to the end of the introduction:

Lines 108-119: “Here we show that K25 is capable of recruiting a second inositol phosphate molecule, in addition to that bound by R18. Sequentially replacing each K25 within the hexamer with alanine leads to a dose dependent decrease in infection and RT. Purified K25A virions are also less able to utilize dNTPs to synthesize DNA in vitro. This data is supportive of a role for K25 in importing dNTPs to allow RT and promote infection. However, analysis of HIV capsids within virions by Electron cryotomography (CryoET) and single molecule total internal reflection fluorescence (TIRF) microscopy reveals that K25 mutants have a severe assembly and stability defect. In vitro assembly experiments confirm that K25 is required for efficient IP6-driven assembly of mature capsid cones. On the basis of these results, we propose that K25 uses IP6 to promote mature capsid assembly. Nevertheless, the inability of those K25A capsids that do assemble to undergo efficient reverse transcription suggests it is also required for dNTP import.”

Reviewer #3

Q1. Thermostability experiments (Fig 3A) seem to suggest that Lys25 may not be critical for IP6 binding to CA hexamers. Based on this, and additional results (specifically the ability of K25A to assemble into tubes but not cones), the authors speculate that the Lys25-IP6 interaction might be more important in the context of the pentamer. A measurement with disulfide-crosslinked pentamers could greatly help support this hypothesis.

A1. We thank the reviewer for this suggestion and have included nanoDSF measurements of disulphide cross-linked pentamers (Fig 6SC). We can show that IP6 does indeed bind to and stabilize pentamers.

Q2. Further to this, does IP6 fit into the pentamer pore without steric problems?

A2. We have modelled IP6 into our IP5 structure as suggested (Figure S1a).

**Part III – Minor Issues: Editorial and Data Presentation Modifications**

Reviewer #1:

Q1. Numerous relevant references are missing. For example, there is no reference to the recent work suggesting that the cores may remain intact till they reach to the nucleus. There is no discussion of what is known of core stability and CA mutations involved in maintaining stability.

A1. We have included the most recent work suggesting that HIV capsids stay intact until they are in the nucleus (Lines 84-87): “Reverse transcription (RT) and, thus, productive infection are closely correlated to capsid stability and its loss, a process called uncoating. [5-7]. Recently it has been shown that the HIV capsid remains largely intact until it enters the nucleus. Reverse transcription is then completed in the nucleus prior to uncoating [8, 9].” We have also included a new section in the discussion on core stability (Lines 370-382).

Q2. Figure 2c: What is the evidence that chimeric viruses actually form as opposed to having a population of fully WT vs. fully mutant viruses? In addition, there is no reference to Fig. 2c in the text.

A2. We understand this concern. We have introduced the following into the results (Lines 161-167): “Previous work has shown that there is a strong correlation between input plasmid ratios and the ratios of the wild-type and mutant CA proteins in viral particles [17]. We observed a similar dose-dependent decrease in the infectivity of K25A chimeric viral particles (Fig 2D) as described for R18G [3]. Infection was broadly maintained in viruses predicted to have at least 4 of 6 lysines at position 25 but substantially reduced in mutants with < 3. This is similar to previous data with R18G and provides further evidence that produced viruses are chimeric, as a mixture of fully wild type and fully mutant viruses would give rise to a linear relationship.”

Q3- Figure 2d: Include P38A or K203A substitution in this figure to control for IP6-independent effects in core stability.

A3. We thank the reviewer for this suggestion. We tried to make P38A cores, but unfortunately could not recover any. This is consistent with previous findings in Forshey, J Virol 2002 and in Eschbach, bioRxiv 2020 that P38A core mutants are too unstable to be purified using a sucrose gradient.

Q4. Figure 2: A figure showing Gag levels/processing in cells and effects of mutations on particle release should be incorporated to this figure.

A4. We have included p24 western blots of gag processing in virions (Supplementary Figure 2C) and of gag processing in cells (Fig 2D). We can show that there are no major gag processing defects. We have also included virus production data as measured by RT activity (Fig S2a&b).

Reviewer #2:

Q1. Title: I am not sure that 'mature assembly' is clear for non-expert. You mean 'mature capsid core'?)

A1. We agree that mature capsid assembly is a better title and have changed it.

Q2. Abstract. The use of IP5 and IP6 sounds a bit random (although later understandable in the text). Maybe an early clarification that both stabilise the capsid core would be very helpful.

A2. We have amended the abstract as follows: “We show that whilst IP5, which can functionally replace IP6, engages an arginine ring (R18) at the top of the pore, the lysine ring simultaneously binds a second IP5 molecule.”

Q3. Final sentence of intro. It is unclear which of the two models the results provided here support (if they support any). I would be more explicit here.

A3. We have followed this suggestion and added an expanded section to the end of the introduction (Lines 108-119).

Q4. Figure 2c should be referenced in the section describing it.

A4. This has been corrected.

Q5. We observed similar dose-dependent decrease [...]. I would clarify that infectivity refers to the purified viral particles.

A5. We have changed to (Line 163): “similar dose-dependent decrease in the infectivity of K25A chimeric viral particles”

Q6. Description of the fluorescent viruses in the main text will be very helpful to understand why and how the fluorescence gets into the particle.

A6. We are not sure whether this refers to the GFP fluorescent viruses or the viruses labelled with fluorescent CypA during TIRF. To clarify the former, we have added (Lines 218-221): “We co-infected cells with a WT virus that encodes a GFP reporter gene and a variety of mutant viruses that encode an RFP reporter gene (for instance, a single infection includes both WT-GFP and K25R-RFP).” For the latter we have added (Lines 241-242): “This enables fluorescent-labelled CypA in bulk solution to pass into the virion through the membrane pore to bind and ‘paint’ any intact capsid lattices that are present.”

Reviewer #3: (No Response)
